# The Dynamic Evolution of Eosinophilic Esophagitis

**DOI:** 10.3390/diagnostics15030240

**Published:** 2025-01-21

**Authors:** Amir Farah, Tarek Assaf, Jawad Hindy, Wisam Abboud, Mostafa Mahamid, Edoardo Vincenzo Savarino, Amir Mari

**Affiliations:** 1 Department of Surgery, Medical College of Wisconsin, Milwaukee, WI 53226, USA; amirfrh89@gmail.com; 2 Department of Surgery, Holy Family Hospital, Nazareth 1601001, Israel; tarek89@gmail.com; 3 The Proteomic Unit, Bnai Zion Medical Center, Haifa 3339419, Israel; jawadhindy@hotmail.com; 4 Cancer Research Center, The Rappaport Faculty of Medicine, Technion, Israel Institute of Technology, Haifa 3525433, Israel; 5 Department of Gastroenterology and Hepatology, Bnai Zion Medical Center, Haifa 3339419, Israel; 6 Department of Surgery, EMMS Nazareth Hospital, Nazareth Hospital, Nazareth 1613101, Israel; wisam_abbud@nazhosp.com; 7 Faculty of Medicine, Bar Ilan University, Ramat Gan 5290002, Israel; 8 Department of Internal Medicine, Meir Medical Centre, Kefar Sava 4428164, Israel; mahamidm020@gmail.com; 9 Division of Gastroenterology, Department of Surgery, Oncology and Gastroenterology, University of Padua, 35122 Padua, Italy; edoardosavarino@gmail.com; 10 Department of Gastroenterology, EMMS Nazareth Hospital, Nazareth Hospital, Nazareth 1613101, Israel

**Keywords:** Eosinophilic esophagitis, diagnosis, management, PPI

## Abstract

Eosinophilic esophagitis (EoE) is a chronic, immune-mediated inflammatory condition of the esophagus characterized by eosinophilic infiltration, and hallmark symptoms of esophageal dysfunction such as dysphagia and food impaction. Over the past three decades, EoE has been recognized as a distinct clinical entity, distinguished from gastroesophageal reflux disease (GERD) through advancements in diagnostic techniques, particularly endoscopy with biopsy. The rising global prevalence of EoE reflects enhanced diagnostic awareness, evolving criteria, and environmental along with lifestyle changes. The etiology of EoE is multifactorial, involving genetic predispositions, immune dysregulation, the gut microbiome, and environmental triggers, including dietary allergens and aeroallergens. Key mechanisms include a type 2 helper T-cell (Th2)-driven immune response, epithelial barrier dysfunction, and genetic variants such as *CAPN14* and *TSLP*. Chronic inflammation leads to tissue remodeling, fibrosis, and esophageal narrowing, contributing to disease progression and complications. Management strategies have evolved to include dietary elimination, proton pump inhibitors, topical corticosteroids, biologics, and endoscopic interventions for fibrostenotic complications. Emerging therapies targeting cytokines such as interleukin (IL)-4, IL-5, and IL-13, alongside novel diagnostic tools like the esophageal string test and Cytosponge, offer promising avenues for improved disease control and non-invasive monitoring. Long-term surveillance combining endoscopic and histological evaluations with biomarkers and non-invasive tools is critical to optimizing outcomes and preventing complications. Future research should address gaps in understanding the role of the esophageal microbiome, refine therapeutic approaches, and develop personalized strategies to improve disease management and patient quality of life.

## 1. Introduction

Eosinophilic esophagitis (EoE) is increasingly recognized as a chronic, immune-mediated type 2 inflammatory condition of the esophagus marked by significant eosinophil infiltration [1]. This inflammation disrupts normal esophageal function and commonly leads to symptoms such as dysphagia (difficulty swallowing), food impaction, and esophageal discomfort [2,3]. First identified as a distinct clinical condition in the early 1990s, EoE was often initially misdiagnosed as gastroesophageal reflux disease (GERD) due to overlapping symptoms [4,5]. However, advancements in diagnostic techniques, particularly endoscopy with biopsy and histological analysis, have clarified EoE as a unique disorder that necessitates specific management strategies distinct from GERD [6,7]. Although they are separate entities, the historical overlap between EoE and GERD cannot be ignored. Features such as the presence of dysphagia and heartburn symptomatology, both may show eosinophilic inflammation to varying degrees on biopsy, as well as their very similar presentation in children, with symptoms such as vomiting, failure to thrive, and abdominal pain [8].

The etiology of EoE is complex and multifactorial, involving genetic predispositions and environmental influences, particularly dietary allergens, and aeroallergens [9,10]. Studies demonstrate a strong association between EoE and atopic conditions, such as asthma, eczema, and food allergies, indicating a shared immunological foundation driven by an overactive type 2 helper T-cell (Th2) immune response [11,12]. Recent genetic research, including genome-wide association studies (GWAS), has identified polymorphisms in genes associated with epithelial barrier integrity and immune response, such as CAPN14 and TSLP, further underscoring the role of genetic susceptibility [3,10,13].

The prevalence of EoE has risen considerably over recent decades, with estimates suggesting that up to 1 in 2000 individuals in the United States may be affected, although figures vary globally [2,14]. This increase likely reflects not only improved diagnostic awareness and criteria but also lifestyle and environmental changes that have enhanced allergen exposure [4,15]. The rising prevalence may also be due to Westernized diets, poor water quality, and high particulate air pollution [16]. Moreover, due to disruptions in the gut microbiome due to maternal antibiotic use during pregnancy, and acid suppression during infancy. Lastly, the rising rates of asthma, allergic rhinitis, eczema, and food allergies parallel the increase in EoE, indicating shared environmental and genetic risk factors [16]. Quality of life is substantially impacted in individuals with EoE, given the chronic nature of the disease and the dietary modifications, pharmacological treatments, and sometimes invasive interventions required for symptom control and complication prevention [6,17,18]. These challenges demonstrate the need for a continued research focus to advance diagnostic techniques, refine treatment protocols, and deepen the understanding of EoE’s complex pathophysiology [4,19]. This literature review aims to address the agreed-upon milestones of this disease, as well as to dive into the challenges and gaps in the literature surrounding it.

## 2. Definition and Historical Context of Eosinophilic Esophagitis

EoE is defined as a chronic, immune-mediated inflammatory condition that affects the esophagus, characterized by a large eosinophilic infiltration leading to esophageal dysfunction [2]. EoE typically manifests with symptoms such as dysphagia, food impaction, and esophageal discomfort, though presentation can vary depending on age and other factors [6,20]. In 1993, Attwood et al. first described EoE as a unique condition distinct from GERD, noting a lack of response to proton pump inhibitors (PPIs) in EoE patients compared to those with GERD [5]. Following this, studies reinforced EoE’s distinctiveness from GERD, leading to its classification as a separate disorder with unique diagnostic and treatment needs [6,20,21].

The current diagnostic criteria for EoE include the presence of at least 15 eosinophils per high-power field (HPF) in esophageal biopsy samples, accompanied by the exclusion of other causes of esophageal eosinophilia, such as GERD, eosinophilic gastroenteritis, and certain infections [22,23]. This standard was established to improve diagnostic accuracy, given the overlap in symptoms with other esophageal and gastrointestinal disorders [4,20]. Notably, the connection between EoE and allergic diseases has fueled research into the immunological mechanisms underpinning the condition, particularly regarding Th2-mediated pathways, which play a central role in many atopic diseases [7,9,24].

Genetic research has also advanced our understanding of EoE. Studies have identified several genetic polymorphisms associated with increased risk of EoE, including variants in CAPN14, which regulates epithelial barrier function and repair, and TSLP, which plays a role in Th2 immune activation [7,13,25]. Studies have also shown that these genetic components may have synergistic effects when present [13]. For some patients with Mendelian syndromes with coexisting EoE, rare genetically mediated variations may occur, and may shed light on generational incidence in this patient cohort [25]. These genetic markers not only deepen our understanding of EoE pathogenesis but also offer potential avenues for personalized therapy in the future.

## 3. Epidemiology

The global incidence and prevalence of EoE have increased significantly over the past decades, reflecting growing awareness, improved diagnostic capabilities, and potential shifts in environmental and lifestyle factors. A systematic review and meta-analysis by Hahn et al. encompassing data from 1976 to 2022 revealed that the global pooled incidence of EoE is 5.31 cases per 100,000 inhabitant-years, while its prevalence is 40.04 cases per 100,000 inhabitant-years [26]. Higher rates of incidence and prevalence were observed in high-income countries, particularly in North America and Europe, compared to low- or middle-income countries [26]. Notably, the prevalence has shown a marked upward trend, rising from 8.18 cases per 100,000 inhabitant-years during the period of 1976–2001 to 74.42 cases per 100,000 inhabitant-years between 2017 and 2022 [26]. This rise is attributed to expanded diagnostic criteria and increased recognition of the disease, along with heightened rates of endoscopic procedures and biopsies. Advancements in diagnostic tools and biopsy techniques for EoE include the development of the EoE Endoscopic Reference Score to standardize and improve the detection of endoscopic findings such as edema, rings, exudates, and furrows. Additionally, recommendations now emphasize obtaining at least six biopsies from multiple esophageal sites, including proximal and distal regions, to account for the patchy distribution of the inflammation. Emerging histological scoring systems such as the EoE Histology Scoring System enable detailed assessment of inflammation and fibrosis, improving diagnostic accuracy [27]. The data emphasize significant geographical and demographic variations, influenced by factors such as socioeconomic conditions, healthcare access, dietary patterns, and genetic predispositions. The increase may also be attributed to trends associated with lifestyle and environmental changes, including increased exposure to allergens [28,29].

Epidemiological studies have demonstrated that EoE is significantly more common in males than females, with a male-to-female ratio of approximately 3:1, and is most frequently diagnosed in children and young adults, although it can affect individuals across all age groups [2,9].

Interestingly, the prevalence of EoE is lower in Asian populations [30]. In Western countries, EoE has been reported in 1 in every 200 endoscopies, while reports from Asia have reported EoE in about 1 in every 5000 endoscopies [30]. These findings also suggest that genetic or environmental factors unique to Western countries may be implicated.

Understanding these epidemiological trends and environmental tendencies is essential for identifying at-risk populations and tailoring prevention and intervention strategies accordingly.

## 4. Pathophysiology of Eosinophilic Esophagitis

The pathophysiology of EoE is complex, involving a combination of genetic predispositions, environmental exposures, and immune system dysregulation. These factors interact to drive a chronic inflammatory process in the esophagus, characterized by eosinophilic infiltration, tissue remodeling, and functional impairment.

### 4.1. Immune System and Inflammatory Response

EoE is primarily mediated by a type 2 helper T-cell (Th2)-driven immune response, which is triggered by food and environmental allergens. Upon allergen exposure, epithelial cells release cytokines such as thymic stromal lymphopoietin (TSLP), interleukin-33 (IL-33), and IL-25, which activate dendritic cells and group 2 innate lymphoid cells (ILC2s). These cells, in turn, secrete Th2-polarizing cytokines, including IL-4, IL-5, and IL-13, amplifying the inflammatory cascade [31,32]. IL-5 is critical for eosinophil proliferation and survival, while IL-13 drives epithelial cell dysfunction and tissue remodeling by upregulating eotaxin-3 (CCL26), which recruits eosinophils to the esophageal mucosa [32].

Epithelial dysfunction in EoE may involve the RipIL-33 pathway, wherein IL-33, released by damaged epithelial cells, heightens innate immune responses [32]. This pathway exemplifies how epithelial damage contributes not only to chronic inflammation but also to sustained Th2 activity. Mast cells, which are increased in EoE, release mediators such as histamine and tryptase, further exacerbating inflammation and remodeling. The involvement of ILC2s, although not yet fully understood, represents an emerging area of interest in the innate immune mechanisms of EoE [33]. In summary, EoE involves a complex interplay between Th2-driven immune responses and epithelial dysfunction, where allergens trigger cytokine release that amplifies inflammation, recruits eosinophils, and drives tissue remodeling.

### 4.2. Epithelial Barrier Dysfunction

Epithelial barrier dysfunction is a hallmark of EoE. Loss of structural proteins, including E-cadherin, claudins, and desmoglein-1 (DSG1), weakens tight junctions and adherens junctions, facilitating allergen penetration and immune activation [33]. IL-13 plays a central role in disrupting epithelial integrity by regulating the expression of CAPN14, a protease specifically upregulated in the esophageal epithelium of EoE patients. CAPN14 contributes to proteolytic activity, exacerbating barrier dysfunction and perpetuating inflammation [10,32].

Additionally, reduced levels of serine peptidase inhibitor Kazal type 7 (SPINK7) were observed in EoE, further promoting protease-mediated epithelial damage [32]. The combined effects of disrupted tight junctions and excessive proteolytic activity underline the critical role of epithelial dysfunction in disease pathogenesis.

### 4.3. Role of Genetics

Genetic susceptibility is a significant contributor to EoE pathophysiology. Variants in the TSLP gene enhance cytokine expression, promoting Th2 immune polarization. CAPN14 polymorphisms are particularly relevant due to the gene’s exclusive expression in the esophagus and its regulation by IL-13 [10,33]. Recent studies have also implicated genetic alterations in filaggrin (FLG), a protein essential for barrier function, in increasing susceptibility to allergen penetration [33].

GWAS have identified additional loci, including those encoding STAT6 and DSG1, linking genetic variability to immune dysregulation and epithelial barrier integrity. While these genetic factors offer insights into disease mechanisms, gaps remain in understanding how these variants interact with environmental exposures to modulate disease severity [32,33].

### 4.4. Environmental Triggers and Allergens

Environmental factors, particularly dietary allergens such as milk, soy, wheat, and eggs, are central to EoE pathogenesis. Aeroallergens, such as pollen, also play a role, as evidenced by seasonal variations in symptom severity. The Western diet, characterized by processed foods and preservatives, may contribute to epithelial barrier dysfunction and increased allergen exposure [33]. Recent evidence suggests that alterations in the esophageal microbiome, including reduced microbial diversity, may influence immune responses and barrier function, though this area requires further study [32].

Dietary and environmental allergens are well-established triggers, yet the molecular mechanisms behind allergen-specific immune activation are still not fully understood. Further research is needed to uncover how specific dietary components react with genetic predispositions and immune pathways to intensify disease progression not only in EoE but also in a wide variety of allergy-driven, multifactorial diseases.

### 4.5. Mechanisms of Esophageal Damage and Remodeling

Chronic inflammation in EoE leads to progressive tissue remodeling characterized by fibrosis, angiogenesis, and smooth muscle hypertrophy. Eosinophils release toxic granules containing major basic protein (MBP) and eosinophil-derived neurotoxin (EDN), which induce epithelial cell apoptosis and nerve dysfunction, contributing to dysphagia [32]. Transforming growth factor-beta (TGF-β1), upregulated in EoE, is a key mediator of fibroblast activation and extracellular matrix deposition, resulting in esophageal stiffening and stricture formation [33].

Emerging evidence also implicates periostin, a matricellular protein upregulated by IL-13, in enhancing fibrosis and tissue remodeling. This protein not only facilitates eosinophil recruitment but also promotes collagen deposition, underscoring its role in the chronicity of EoE [32].

Despite advancements, significant areas remain unexplored in the relationship between genetic, immune, and environmental factors in EoE. The role of the microbiome in disease onset and progression is limited, as is the contribution of innate immune cells like ILC2s to chronic inflammation [34,35]. Additionally, while biomarkers such as TSLP and periostin hold promise, their utility in predicting disease severity and treatment response requires further validation. As such, future research should focus on identifying additional genetic loci associated with disease heterogeneity, investigating the role of the esophageal microbiome and its therapeutic modulation, and further understanding the molecular mechanisms of allergen-specific immune responses, and developing non-invasive biomarkers for monitoring the disease’s activity and to predict fibrosis [36].

## 5. Clinical Presentation and Diagnosis

EoE manifests with a diverse range of symptoms that vary depending on the patient’s age, disease stage, and comorbid conditions. Adults frequently present with esophageal dysfunction symptoms, such as dysphagia to solid foods and food impaction, which are considered hallmark indicators of the disease [2,3,4]. Studies have revealed that food impaction is one of the most common emergency presentations of EoE in adults, often prompting the first diagnostic investigation [37,38]. In contrast, children may exhibit nonspecific symptoms, including feeding difficulties, vomiting, and abdominal pain, which often overlap with other gastrointestinal conditions and delay diagnosis [20,21].

The variability of symptoms in children versus adults is attributed to differences in disease progression and adaptive behaviors. Pediatric patients may adapt to esophageal dysfunction by avoiding certain textures or displaying failure to thrive, while adults often delay seeking care, adapting through prolonged chewing or liquid ingestion to ease swallowing [39]. These adaptations may obscure the underlying condition, leading to diagnostic delay or misdiagnosis.

In addition to the variability in symptoms, patients with EoE may exhibit seasonal exacerbations, particularly in regions with high aeroallergen exposure. This suggests a role for environmental triggers alongside dietary factors in driving disease activity. However, the specific mechanisms by which seasonal aeroallergens exacerbate EoE remain unclear, identifying a gap in the current understanding of disease pathophysiology [40].

### 5.1. Diagnostic Approach

EoE diagnosis requires a comprehensive, multidisciplinary approach, incorporating clinical history, endoscopic findings, and histological evaluation. Accurate diagnosis is particularly challenging due to the overlap of EoE with other esophageal conditions, such as GERD and eosinophilic gastroenteritis. Misdiagnosis or delayed diagnosis often results in disease progression to advanced stages characterized by esophageal remodeling, fibrosis, and strictures [39]. The diagnosis of EoE requires a systematic approach, the American Gastroenterological Association (AGA) and the Joint Task Force (JTF) on Allergy-Immunology Practice Parameters Clinical Guidelines emphasize a stepwise diagnostic algorithm that includes ruling out secondary causes of eosinophilia, such as GERD or eosinophilic gastroenteritis, through clinical history, histology, and esophageal pH testing [41]. Greuter and Straumann [42] further emphasized the role of high-resolution manometry and barrow swallow in atypical cases or when suspected motility disorders coexist with EoE-like symptoms. Additionally, novel diagnostic tools such as the EndoFLIP, esophageal string test, and Cytosponge are emerging, but their clinical utility remains under evaluation.

### 5.2. Endoscopic Features

**Figure 1:**
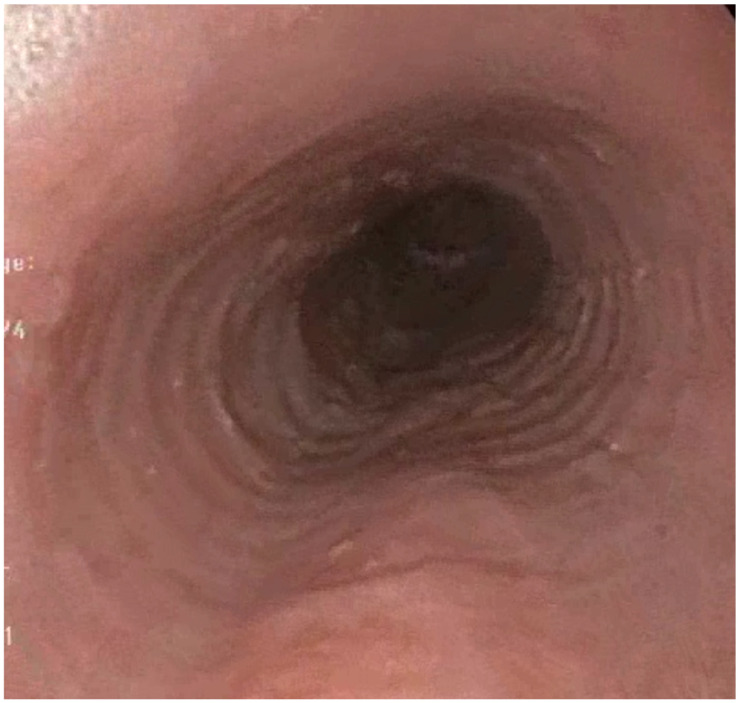
Endoscopic view of the esophagus with active EoE showing rings and longitudinal furrows.

Endoscopy plays a central role in the diagnostic process. (Figure 1) The EREFS (Edema, Rings, Exudates, Furrows, Stenosis) scoring system has standardized the reporting of characteristic endoscopic findings associated with EoE. Studies indicate that linear furrows and white exudates are the most predictive of eosinophilic infiltration and active inflammation, while concentric rings and strictures often reflect chronic remodeling [3,39]. However, up to 11% of patients may have a normal-appearing esophagus, underscoring the importance of histological confirmation regardless of endoscopic findings [39]. Other limitations of the EREFS scoring system include a limited sensitivity for fibro-stenotic features such as strictures, variability in scoring across endoscopists, and a lack of direct correlation with symptoms [43].

Technological advancements such as high-resolution imaging and chromoendoscopy are being evaluated to enhance the detection of subtle mucosal changes indicative of EoE. However, their cost and availability limit widespread adoption, particularly in resource-constrained settings [40].

### 5.3. Histological Evaluation

**Figure 2:**
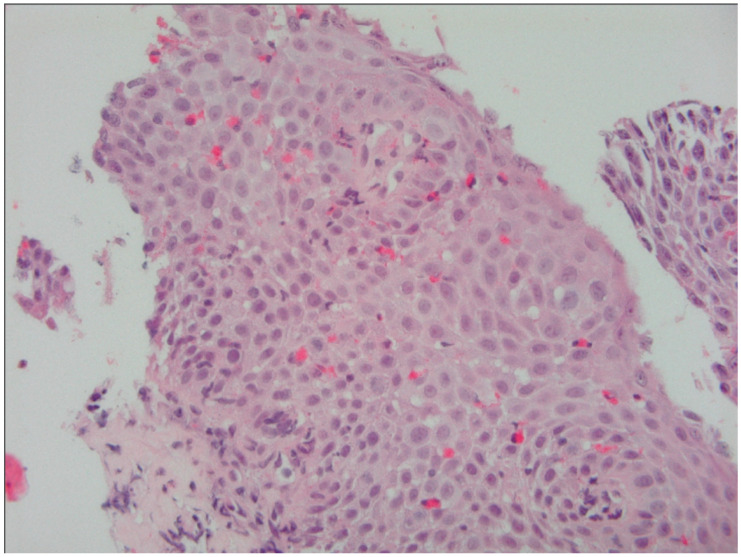
Esophageal biopsy of a patient with active EoE showing infiltration of numerous eosinophils in the mucosa.

Histological analysis remains the diagnostic gold standard for EoE. (Figure 2) Current guidelines define EoE by the presence of at least 15 eosinophils per high-power field (HPF) in esophageal biopsies, taken from multiple esophageal regions to account for the patchy nature of eosinophilic infiltration [14,22]. Other histological features, such as basal layer hyperplasia, elongation of the papillae, and lamina propria fibrosis, offer additional insight into disease chronicity and severity [39].

Emerging histological scoring systems aim to quantify inflammation and tissue remodeling more precisely. These tools have demonstrated potential for assessing treatment efficacy and predicting disease progression, particularly in patients with refractory symptoms or advanced disease [39].

### 5.4. The Role of Manometry in EoE

Although it is not a primary diagnostic criterion, manometry offers functional information that complements endoscopic and histological findings, particularly in cases where overlapping esophageal disorders complicate diagnosis [39,44,45,46].

Esophageal motility disturbances are frequently observed in EoE, arising from chronic inflammation and tissue remodeling. High-resolution manometry (HRM) studies have identified patterns such as fragmented peristalsis, ineffective esophageal motility (IEM), and reduced distal contractile integral (DCI), all of which correlate with the extent of inflammation and fibrosis [40,44]. Reduced DCI, in particular, is associated with the fibrostenotic phenotype of EoE, reflecting impaired esophageal compliance and muscle contraction [47]. The HIMEOS study, which examined esophageal motility patterns across diverse phenotypes of EoE, emphasized the variability in hypomotility and impaired bolus transit in relation to disease chronicity, further validating manometry as a tool to stratify disease stages and guide management [47]. Impaired peristaltic coordination in EoE is thought to arise from structural alterations, such as subepithelial fibrosis, that disrupt neuromuscular signaling in the esophageal wall. Additionally, transient pressurization patterns observed during HRM, particularly panesophageal pressurization, are indicative of reduced esophageal distensibility and stiffness caused by fibrosis [44].

The inclusion of impedance technology alongside manometry enhances the diagnostic value of HRM by assessing bolus transit efficiency, which is often impaired in EoE. Studies indicate that impedance manometry identifies incomplete bolus clearance in patients with severe inflammation and esophageal narrowing, offering a functional correlation to endoscopic and histological findings [39,44]. Impedance findings of impaired bolus transit often align with the presence of esophageal strictures or rings, accentuating their role in evaluating mechanical and motility-related burdens of EoE [47]. This approach broadens the scope of manometry by capturing both transit and contractile deficits, providing a more nuanced assessment of disease severity and functional limitations.

Manometry also plays a significant role in distinguishing EoE from other esophageal motility disorders, such as achalasia and distal esophageal spasm (DES). Achalasia, characterized by impaired lower esophageal sphincter (LES) relaxation and absent peristalsis, is rarely observed in EoE, whereas hypercontractile activity in DES contrasts with the weak or fragmented peristalsis seen in EoE [39,44,45,46]. HRM findings in EoE frequently include hypocontractile patterns, a marked departure from the hypercontractility typical of DES, further aiding differentiation. Additionally, manometry can differentiate EoE from GERD by revealing esophageal hypomotility patterns and impaired bolus transit in EoE, features that are less pronounced in GERD and more indicative of inflammatory or fibrostenotic changes [44,47]. These distinctions highlight the utility of manometry in clarifying diagnostic uncertainty and optimizing disease management.

Emerging evidence supports the utility of advanced manometric protocols, such as provocative maneuvers, in detecting subtle motility abnormalities in EoE. Tests such as multiple rapid swallows (MRS) and solid swallows can reveal increased intrabolus pressure (IBP) and transient pressurization, which correlate with symptom severity and esophageal remodeling [44]. For example, elevated IBP identified during MRS may indicate esophageal outflow obstruction or reduced compliance associated with fibrotic changes, offering a functional biomarker of disease progression [47]. These methods provide deeper insights into esophageal dysfunction beyond standard manometric assessments, potentially refining diagnostic accuracy and tailoring therapeutic interventions.

Despite its advantages, manometry is not yet fully integrated into routine diagnostic protocols for EoE, partly due to variability in findings and the absence of disease-specific motility signatures. The heterogeneity in manometric profiles across different disease stages—ranging from reversible motility disturbances in inflammatory-phase EoE to persistent hypomotility in fibrostenotic phenotypes—presents a significant challenge [44]. Furthermore, the lack of consensus on specific manometric criteria for EoE limits its broader adoption in clinical practice. Future research should prioritize standardizing manometric protocols and incorporating them into a multidisciplinary diagnostic framework to enhance their utility in clinical decision-making.

### 5.5. Emerging Diagnostic Tools

Emerging diagnostic modalities are addressing limitations associated with traditional methods. Non-invasive techniques, such as the esophageal string test and cytosponge devices, have shown promise for monitoring eosinophilic activity without requiring repeated endoscopy. These methods are particularly beneficial for pediatric populations, where repeated endoscopic procedures pose greater risks and logistical challenges [39].

Serum biomarkers, including eotaxin-3 and periostin, are under investigation as potential adjuncts to endoscopic and histological assessments [36,40]. While preliminary studies indicate their utility in tracking disease activity, further validation is needed to integrate these biomarkers into routine clinical practice.

### 5.6. Differentiating EoE from GERD

A significant diagnostic challenge in EoE is distinguishing it from GERD, given the overlap in clinical and endoscopic features. The response to PPI has traditionally been used as a differentiating factor, with EoE typically showing no response. However, recent evidence suggests that PPI-responsive esophageal eosinophilia (PPI-REE) shares histological and molecular characteristics with EoE, further complicating differentiation [39]. Advanced molecular profiling and histological biomarkers may offer new avenues for distinguishing these entities more effectively. Reflux testing, particularly impedance-pH monitoring, plays an important role in distinguishing EoE from GERD by assessing both the reflux burden and esophageal mucosal integrity. In the context of EoE, patients often exhibit reduced baseline mucosal impedance, correlating with mucosal damage and eosinophilia. Impedance-pH testing allows for the evaluation of chemical clearance metrics, such as the post-reflux swallow-induced peristaltic wave (PSPW) index and mean nocturnal baseline impedance (MNBI). These metrics differentiate PPI-responsive from PPI-refractory EoE, aiding in identifying cases where reflux contributes to the disease pathology. Thus, reflux testing serves as a diagnostic adjunct to histology and endoscopic findings, improving accuracy in distinguishing EoE from GERD and guiding effective treatment strategies [48,49].

## 6. Management

The management of EoE requires a multidisciplinary and individualized approach that may include dietary modifications, pharmacologic treatments, and endoscopic interventions. Each of these strategies aims to reduce eosinophilic inflammation, alleviate symptoms, and prevent complications such as fibrosis and stricture formation [50].

### 6.1. Dietary Modifications and Elimination Diets

Dietary therapy remains the first-line management for many EoE patients due to its direct targeting of food allergens implicated in the disease pathogenesis. Studies across diverse patient cohorts solidify its effectiveness but also reveal challenges in adherence and patient burden [51].

Six-Food Elimination Diet (SFED): The SFED eliminates the six most common allergens—milk, wheat, eggs, soy, nuts, and seafood, and has achieved histological remission in 72–74% of patients in some studies [52]. However, long-term adherence is challenging, and patients often require close dietary supervision to prevent nutritional deficiencies. Research suggests that strict adherence to SFED for at least six weeks is necessary to observe significant remission, and subsequent food reintroduction protocols can help identify triggers while maintaining symptom control [53]. The AGA approaches suggest a step-up or step-down dietary strategy. The SFED is often initiated to identify food triggers, followed by gradual reintroduction to pinpoint allergens while reducing dietary restrictions. Alternatively, less restrictive strategies, such as two or four-food elimination diets may improve adherence and quality of life while maintaining efficacy [41].

Four-Food Elimination Diet (FFED): A more flexible alternative, the FFED excludes dairy, wheat, eggs, and soy, with remission rates reported between 54% and 60% in prospective studies [15]. The FFED demonstrates improved adherence and nutritional outcomes compared to SFED and is particularly favored in pediatric populations [54,55]. In a prospective study of pediatric patients with EoE, 2 months of FFED resulted in clinical, endoscopic, and histologic remission in more than 60% of children with EoE, which was nearly as effective yet less restrictive than SFED [56].

One-Food Elimination Diet (OFED): Typically involves the exclusion of animal milk, and plays a significant role in the management of EoE [57,58]. Studies have shown that cow’s milk is a common dietary trigger for esophageal eosinophilia in both adults and children [57,58]. OFED is less restrictive compared to multi-food elimination diets and has demonstrated efficacy in achieving histologic remission in approximately 50–55% of patients [58]. This approach offers an easier-to-implement alternative that may lead to better adherence while minimizing dietary restrictions [57,58]. By focusing on the exclusion of a single highly implicated allergen, OFED balances efficacy with patient quality of life, positioning it as an initial dietary therapy option for managing EoE [58].

Elemental Diet: The elemental diet, a highly restrictive dietary intervention, has shown significant efficacy in the management of EoE. By substituting all food intake with an amino acid-based formula, this approach eliminates exposure to potential food allergens, resulting in marked reductions in esophageal eosinophilic inflammation. Studies by Peterson et al. and Warners et al. confirm the histological remission rates of approximately 72% and 71%, respectively, in adults, with additional improvements in endoscopic inflammatory signs and symptoms [59,60].

Warners et al. documented significant reductions in peak eosinophil counts, from 40 to 9 eosinophils per HPF, following a four-week elemental diet [60]. Similarly, Peterson et al. observed reductions from 54 to 10 eosinophils per HPF, with histological responses evident as early as two weeks into treatment [59]. The studies underscore the diet’s ability to mitigate both eosinophil counts and associated mast cell activity, key contributors to EoE pathophysiology.

Despite its efficacy, the elemental diet poses substantial challenges. Both studies highlight poor adherence due to palatability issues, with drop-out rates of 38% and 19%, respectively. Warners et al. emphasized that the inclusion of improved formulas with varied flavors and regular monitoring by dieticians enhances compliance [60]. However, significant weight loss remains a common concern, with Peterson et al. reporting losses exceeding 7 kg in some cases [59].

The elemental diet is particularly effective in inducing rapid remission of inflammatory features, including furrows and exudates; however, fibrostenotic changes such as strictures and rings are less responsive, suggesting a limited role in reversing advanced disease remodeling [60].

Given its efficacy, the elemental diet is often reserved for refractory cases or as a diagnostic tool to identify dietary triggers in reintroduction protocols. Both studies reinforce its role as a potent but demanding therapeutic option, requiring meticulous adherence strategies to balance clinical benefits against patient burden. Further research into alternative formulations and long-term outcomes is necessary to optimize its utility in EoE management.

Emerging strategies to improve dietary therapy outcomes include allergy testing-based elimination diets and hybrid approaches combining empirical elimination with targeted food reintroductions. Studies indicate that identifying patient-specific triggers through reintroduction improves adherence and reduces dietary restrictions [23,53].

### 6.2. Pharmacological Treatments

Pharmacologic therapy becomes essential in managing EoE in patients unable to adhere to dietary therapy, those with severe inflammation, or for refractory symptoms. The evolving pharmacologic landscape includes proton pump inhibitors, corticosteroids, and biologic agents.

Proton Pump Inhibitors: Initially prescribed for GERD, PPIs are now recognized for their anti-inflammatory effects in EoE. Approximately 50% of patients demonstrate remission on PPI therapy, which inhibits IL-13-induced eotaxin-3 production and reduces eosinophilic recruitment [15]. In a study by Navarro et al., an additional therapeutic benefit of PPI in EoE beyond anti-inflammatory effects. PPIs were shown to significantly reduce endoscopic fibrosis, measured via the EREFS scoring system, particularly in features like rings and strictures, after a short treatment course [61]. While PPIs demonstrated a 50% clinico-histological remission rate, their impact on reducing fibrotic remodeling solidifies their role in potentially reversing structural esophageal damage associated with long-term EoE. This contrasts with swallowed corticosteroids, which, while more effective in achieving deep histological remission, showed comparable efficacy in improving esophageal compliance and reducing fibrotic features. However, the dual role of PPIs in treating both EoE and GERD may complicate diagnoses, as PPIs can suppress eosinophilic inflammation in EoE, masking histological signs when biopsies are taken during investigations. To avoid diagnostic masking, PPI should be withdrawn 3–4 weeks before biopsies, though this can exacerbate GERD symptoms [27,40]. Studies further elucidate the role of PPIs in managing EoE. A cross-sectional analysis of the EoE CONNECT database assessed the effectiveness of PPI therapy in real-world settings. Among 630 patients, PPI therapy achieved histological remission (eosinophil density below 15 eos/hpf) in 48.8% and a significant reduction in symptom scores in 71% of patients. Factors such as an inflammatory phenotype and treatment duration for up to 12 weeks were associated with improved clinico-histological outcomes, while a stricturing phenotype negatively impacted efficacy. Importantly, sustained clinical remission was maintained with dose reduction in 69.9% of patients [62].

Additionally, a study evaluating the effects of PPIs on fibrotic features in EoE revealed that short-term PPI therapy reduced endoscopic signs of fibrosis, such as rings and strictures, as measured by the EREFS score. Clinico-histological remission was achieved in 50% of patients, with deep histological remission (<5 eos/hpf) observed in 36%. These findings underscore the potential of PPIs not only in reducing inflammation but also in reversing fibrotic remodeling associated with chronic EoE [62].

Data also show that high-dose PPIs (e.g., esomeprazole 40 mg twice daily) are required to achieve optimal results, with symptomatic improvement observed within four to eight weeks of treatment [53,63].

Topical Corticosteroids: Topical corticosteroids remain the cornerstone of pharmacological therapy for EoE, a chronic, immune-mediated disease characterized by esophageal dysfunction and eosinophil-predominant inflammation [40,64]. Topical corticosteroid are primarily employed to induce histological and symptomatic remission by targeting localized inflammation while minimizing systemic side effects [64].

Budesonide orally disintegrating tablets (BOT) and oral viscous budesonide (OVB) are among the most effective formulations, with BOT achieving histological remission rates exceeding 80% in clinical trials [64,65]. In a randomized controlled trial involving 88 patients, BOT 1 mg twice daily achieved clinical and histological remission in 57.6% of patients, compared to 0% in the placebo group (*p* < 0.0001) [66]. In a subsequent maintenance study, BOT maintained persistent remission in 75.0% and 73.5% of patients using 1 mg and 0.5 mg doses twice daily, respectively, compared to 4.4% in the placebo group at week 48 (*p* < 0.001) [65].

Oral budesonide suspension (BOS) has similarly demonstrated efficacy, with an RCT on 318 patients showing histological remission in 53.1% of patients receiving BOS 2 mg twice daily, compared to 1.0% with placebo (*p* < 0.001). Clinical remission rates were also significantly higher in the BOS group (52.6% vs. 39.1%; *p* = 0.02) [65]. In a maintenance study, BOS 2 mg daily maintained remission in 83.3% of patients at week 36, compared to 50.0% with placebo (*p* = 0.03).

A recent network meta-analysis confirmed that approved therapies, including BOT 1 mg twice daily and BOS 1 mg and 2 mg twice daily, are significantly more effective than placebo for inducing histological remission (defined as <15 eos/HPF) [64,65].

In terms of symptom improvement, BOT 1 mg and BOS 2 mg twice daily ranked highest, while BOT and BOS also led in improving endoscopic findings based on the EREFS score [65]. However, challenges persist, including risks such as esophageal candidiasis, variable adherence, and the recurrence of inflammation upon treatment cessation [40,65].

These limitations underscore the need for individualized maintenance strategies, guided by endoscopic and histological findings.

The AGA and JTF management algorithm recommends initiating pharmacological treatment with high dose PPIs or swallowed topical corticosteroids as first-line therapies [41]. Induction doses should be maintained for six weeks, with an extension to 12 weeks in cases of incomplete response. Swallowed topical corticosteroids are highlighted as the most potent agents for inducing remission, especially in severe cases. Maintenance therapy, often involving half the induction dose, is crucial to prevent relapse [41].

Biologic Therapies:

Dupilumab, a monoclonal antibody that inhibits the interleukin-4,13 receptor alpha subunit, has demonstrated significant efficacy in the management of EoE, particularly in patients with coexisting atopic conditions such as asthma and allergic rhinitis [64]. In a randomized controlled trial (RCT) of 81 patients, dupilumab administered at 300 mg weekly achieved histological remission in 60% of patients after 24 weeks, compared to 5% in the placebo group (*p* < 0.001) [65]. Notably, dupilumab’s effectiveness is particularly pronounced in patients with a history of type 2 inflammation, such as those with asthma or atopic dermatitis, as the drug targets the underlying inflammatory pathways common to these diseases [65]. In the maintenance phase, efficacy was sustained, with clinical and histological improvements maintained or even enhanced at week 52 [65].

Recent network meta-analyses further support dupilumab’s role in EoE management, confirming its superior efficacy over placebo and placing it on par with other approved treatments like budesonide orally disintegrating tablets and oral budesonide suspension [64].

Additionally, the 1st EoETALY Consensus recommends dupilumab as a second-line treatment option for patients with EoE who fail to respond to first-line therapies such as proton pump inhibitors or topical steroids, particularly those with significant atopic comorbidities [40].

However, despite its promising therapeutic potential, dupilumab is associated with several potential adverse effects, including nasopharyngitis, injection site reactions, conjunctivitis, and an increased risk of infections, all of which require careful monitoring [65].

While the current evidence is compelling, further long-term studies are needed to refine patient selection criteria and assess the drug’s safety profile in broader clinical populations.

From a cost perspective, dupilumab represents a significant financial burden due to its high price, which may limit accessibility for some patients. Although cost-effectiveness analyses have demonstrated its value in severe asthma compared to other biological therapies, affordability remains a concern, particularly in healthcare systems with limited budgets or in populations without insurance [67].

Within biologic therapy, dupilumab is a monoclonal antibody that inhibits IL-4 and IL-13 signaling by blocking the IL-4 receptor alpha. It is the first FDA-approved biologic therapy for EoE, indicated for patients aged 12 and older weighing at least 40 kg. Clinical trials demonstrate that dupilumab significantly reduces eosinophil counts and improves histologic, endoscopic, and symptomatic outcomes. In a phase 3 trial, histologic remission (≤6 eosinophils per high-power field) was achieved in 60% of patients receiving weekly dupilumab, compared to 5% in the placebo group. Symptom improvements were also substantial, particularly in reducing dysphagia scores [68,69]. Adverse effects are generally mild, with injection site reactions and nasopharyngitis being the most common. Dupilumab is recommended as a step-up therapy for refractory cases or for patients with concomitant atopic conditions, such as asthma or dermatitis.

### 6.3. Endoscopic Dilation

Esophageal dilation is a therapeutic option for managing fibrostenotic complications in EoE, particularly for patients presenting with significant strictures or dysphagia unresponsive to medical and dietary therapy. This mechanical approach aims to alleviate esophageal narrowing by increasing luminal diameter, thus improving food passage, and reducing the risk of food impaction.

Studies demonstrate that dilation effectively relieves dysphagia symptoms, with response rates ranging from 85% to 95% across various patient cohorts [70,71]. It achieves these outcomes by mechanically disrupting fibrotic tissue in the esophageal wall, which contributes to luminal narrowing. Over multiple sessions, gradual increases in esophageal diameter, typically aiming for ≥15 mm, can provide sustained symptomatic relief [70]. Both bougie and through-the-scope balloon dilation techniques are utilized, with comparable efficacy and safety profiles. Bougie dilation is particularly advantageous in diffusing esophageal narrowing, whereas balloon dilation allows for targeted management of focal strictures [71].

Endoscopic dilation does not address the underlying eosinophilic inflammation or prevent further fibrotic remodeling, necessitating concurrent anti-inflammatory treatment to mitigate disease progression [70]. Importantly, while the risk of major complications, such as perforation, is minimal (reported at approximately 0.38%) and patients may experience transient post-procedural chest pain in up to 75% of cases, emphasizing the need for cautious dilation protocols [70,71]. Notably, the severity of fibrostenosis, determined by luminal diameter and stricture length, is a predictive factor for symptomatic recurrence and the need for repeat dilation [72].

In addition to its therapeutic benefits, dilation outcomes accentuate the chronicity of the fibrostenotic phenotype in EoE. Patients with longstanding disease often require multiple dilation sessions over time, with up to 58% of individuals needing repeated interventions, frequently within the first year following the initial procedure [70]. The need for repeated dilations is often linked to inadequate control of inflammation, for this reason, it is important to integrate endoscopic interventions with dietary and pharmacologic treatments [72]. These findings identify the importance of early diagnosis and intervention to prevent irreversible esophageal remodeling.

The safety and tolerability of dilation have improved substantially with advancements in endoscopic techniques and procedural protocols. A recent meta-analysis confirmed the low complication rates associated with the procedure, reinforcing its role as a key management strategy in fibrostenotic EoE [71]. Patient-reported outcomes are positive following dilation, with many patients reporting improvement in quality of life, with reduced dysphagia-related anxiety and increased confidence in eating a broad range of foods [72].

### 6.4. Novel and Emerging Therapies

Biologic therapies have transformed the management of EoE by specifically targeting immune pathways implicated in the disease pathogenesis. These treatments are particularly promising for patients with refractory disease or those unable to tolerate conventional therapies. Emerging biologics focus primarily on neutralizing key cytokines like interleukin (IL)-4, IL-5, and IL-13, which are central to the type 2 immune response in EoE.

#### 6.4.1. IL-5 Antagonists

Biologics targeting IL-5, such as mepolizumab and benralizumab, aim to reduce eosinophil recruitment and activation. Mepolizumab binds to IL-5, preventing it from interacting with its receptor, while benralizumab depletes eosinophils by inducing antibody-dependent cell-mediated cytotoxicity. Studies indicate that these agents effectively lower eosinophil counts and may improve symptoms, though results for endoscopic and histologic remission are inconsistent [69]. They are generally well-tolerated, but further large-scale trials are required to establish their role in routine EoE management.

#### 6.4.2. IL-13 Inhibitors

Targeting IL-13 has shown promise in reducing inflammation and esophageal remodeling. Tralokinumab and lebrikizumab, IL-13-specific inhibitors like cendakimab [73], have demonstrated potential in phase 2 trials. Both drugs significantly reduced eosinophil infiltration and improved histologic outcomes. However, endoscopic and symptomatic benefits require further validation in larger, more diverse populations [69].

#### 6.4.3. TSLP and JAK Inhibitors

Thymic stromal lymphopoietin (TSLP) is an upstream cytokine critical for initiating type 2 inflammation in EoE. Tezepelumab, a TSLP antagonist, has shown promise in early trials, particularly in reducing eosinophilic inflammation and symptoms in patients with refractory disease. Janus kinase (JAK) inhibitors, such as tofacitinib, are also being explored due to their ability to inhibit downstream signaling from multiple cytokines involved in the disease process. Preliminary data suggest that JAK inhibitors may reduce eosinophil counts and improve esophageal compliance, but their use is currently limited by safety concerns, including risks of thrombosis and immunosuppression [64,69].

In a systematic review and meta-analysis by Visaggi et al., comparing biologic therapies for EoE, the superior efficacy of dupilumab for achieving histologic and symptomatic remission was established [64]. Among other biologics, tralokinumab and mepolizumab ranked lower in terms of consistent efficacy. The analysis pointed out the need for standardized outcome measures across trials to better compare treatment options [64].

Despite the positivity surrounding the use of biologic therapies, several challenges remain. High costs and the need for long-term therapy may limit widespread use. Additionally, more research is needed to identify biomarkers predictive of treatment response and to understand the long-term safety of these agents. Combination therapies and tailored approaches based on patient-specific immune profiles represent promising avenues for future exploration.

### 6.5. Long-Term Assessment

Given the chronic and relapsing nature of EoE, effective long-term management is crucial to control symptoms, prevent complications, and maintain quality of life for affected patients. As stated, management often involves ongoing dietary restrictions, pharmacologic therapy, and periodic monitoring through endoscopic and histological evaluations [2,29]. Studies, such as those from the EoE CONNECT registry, emphasize the importance of regular follow-ups to track disease progression and assess treatment response, given the variable nature of EoE [74].

Long-term surveillance is an important element in the effective management of EoE given its chronic, relapsing nature and potential for long-term complications such as fibrosis, strictures, and reduced esophageal compliance. Surveillance strategies aim to monitor treatment efficacy, detect disease progression, and prevent complications while minimizing unnecessary interventions.

Endoscopy with biopsy remains the foundation of long-term surveillance in EoE. Studies emphasize that histological remission correlates strongly with improved patient outcomes and a reduced risk of fibrostenotic complications [75]. Periodic endoscopic evaluations are particularly important for patients with persistent symptoms, as endoscopic appearances alone are insufficient to detect subclinical inflammation [76]. Repeat endoscopy is generally recommended 6–12 months after initiating treatment and at regular intervals for maintenance monitoring.

Emerging non-invasive tools offer promising adjuncts to traditional surveillance. Biomarkers such as serum periostin and eosinophil progenitor levels have shown potential in identifying active disease and tracking response to therapy. Henderson et al. demonstrated that elevated eosinophil progenitor levels correlate with histologic activity, making them a valuable surrogate for invasive procedures in certain patient populations [75]. Additionally, the esophageal string test and Cytosponge devices are under evaluation as non-invasive options for monitoring mucosal eosinophilia. The esophageal string test (EST) and Cytosponge are minimally invasive tools for monitoring EoE, offering alternatives to traditional endoscopy [77]. The EST collects esophageal secretions via a nylon string and quantifies eosinophil-associated proteins (e.g., eotaxin-3, MBP-1), correlating with disease activity and treatment response [77]. It is practical, cost-effective, and preferred by patients, particularly children, due to its non-invasive nature and ability to distinguish active from inactive disease.

The Cytosponge, a capsule that expands into a sponge, retrieves epithelial cells for histological analysis, capturing data on inflammation and fibrosis [77]. It offers broader cytological details compared to the EST but faces challenges in pediatric populations.

Both tools may be potentially used for long-term surveillance of EoE by reducing the need for repeated endoscopies, improving patient compliance, and providing critical data on disease progression and treatment outcomes. Their integration into clinical practice could optimize non-invasive disease monitoring and enhance personalized management strategies. More studies are needed to truly validate their role in the surveillance of EoE.

Patients with a fibrostenotic phenotype require intensified surveillance to monitor for recurrence of strictures, even after dilation. Regular endoscopic assessments are crucial for early detection of recurrent narrowing and evaluation of treatment efficacy. A study by Shukla-Udawatta et al. urged the need for individualized follow-up schedules based on disease phenotype, treatment response, and symptom recurrence [76].

Current guidelines advocate a personalized approach to surveillance, incorporating clinical, endoscopic, and histologic assessments. For patients achieving sustained remission, surveillance intervals may be extended, while those with active inflammation or complications require closer monitoring. Multidisciplinary care and adherence to consensus guidelines were shown to reduce variability in follow-up practices and improve outcomes [75,76]. However, variability in institutional protocols highlights the need for further research to standardize long-term monitoring approaches.

## 7. Conclusions

EoE is a chronic, immune-mediated condition with complex etiology and multifaceted management needs. Over the past three decades, advancements in diagnostic precision, particularly through endoscopy and histological evaluation, have distinguished EoE as a unique clinical entity. Epidemiological studies reveal its increasing prevalence, likely influenced by improved awareness, diagnostic criteria, and environmental changes. The disease’s pathophysiology highlights intricate interactions between genetic predispositions, immune dysregulation, and environmental triggers, with significant roles for Th2-mediated inflammation and epithelial barrier dysfunction.

Clinical presentation varies by age, complicating timely diagnosis, but standardized scoring systems and innovative diagnostic tools, such as impedance technology and biomarkers, are improving detection. Emerging diagnostic modalities, including the esophageal string test and Cytosponge, promise to reduce the invasiveness of monitoring, particularly in pediatric populations, although further validation is needed. Addressing the variability in diagnostic and surveillance approaches across institutions is essential to ensure equitable access to consistent, high-quality, and standardized care.

Management strategies for EoE have evolved to incorporate dietary, pharmacological, and endoscopic interventions. Dietary therapy remains a cornerstone, while topical corticosteroids and biologic agents such as dupilumab are expanding therapeutic options, especially for refractory cases. Endoscopic dilation addresses fibrostenotic complications but emphasizes the need for concurrent anti-inflammatory therapy to prevent recurrence. Novel therapies targeting cytokines and pathways involved in eosinophilic inflammation and remodeling offer hope for improved disease control but their cost and long-term safety require careful consideration.

Long-term surveillance is critical for managing EoE’s chronic and relapsing nature, with periodic endoscopic and histological evaluations forming the backbone of monitoring. Non-invasive tools and biomarkers are emerging as valuable adjuncts to traditional surveillance, reducing patient burden and improving compliance. Personalized surveillance protocols based on disease phenotype, treatment response, and symptom severity can optimize outcomes and minimize complications.

Despite these advancements, significant gaps remain. Understanding the molecular mechanisms of allergen-specific immune responses, the role of the microbiome, and the interplay between genetic and environmental factors warrants further exploration. Additionally, standardizing diagnostic and surveillance practices across institutions and validating novel tools are essential for advancing patient care. As the field continues to evolve, a multidisciplinary, individualized approach remains pivotal in improving the quality of life and long-term outcomes for patients with EoE.

## Data Availability

The original contributions presented in the study are included in the article, further inquiries can be directed to the corresponding author.
